# Predictors and nomogram of in-hospital mortality in sepsis-induced myocardial injury: a retrospective cohort study

**DOI:** 10.1186/s12871-023-02189-8

**Published:** 2023-07-07

**Authors:** Kai-Zhi Xu, Ping Xu, Juan-Juan li, A-Fang Zuo, Shu-Bao Wang, Fang Han

**Affiliations:** Emergency and Critical Care Center, Intensive Care Unit, Zhejiang Provincial People’s Hospital, Affiliated People’s Hospital, Hangzhou Medical College, Hangzhou, 310000 China

**Keywords:** Sepsis, Sepsis-induced myocardial injury, Nomogram, 28-day mortality, Troponin T

## Abstract

**Background:**

Sepsis-induced myocardial injury (SIMI) is a common organ dysfunction and is associated with higher mortality in patients with sepsis. We aim to construct a nomogram prediction model to assess the 28-day mortality in patients with SIMI. .

**Method:**

We retrospectively extracted data from Medical Information Mart for Intensive Care (MIMIC-IV) open-source clinical database. SIMI was defined by Troponin T (higher than the 99th percentile of upper reference limit value) and patients with cardiovascular disease were excluded. A prediction model was constructed in the training cohort by backward stepwise Cox proportional hazards regression model. The concordance index (C-index), area under the receiver operating characteristics curve (AUC), net reclassification improvement (NRI), integrated discrimination improvement (IDI), calibration plotting and decision-curve analysis (DCA) were used to evaluate the nomogram.

**Results:**

1312 patients with sepsis were included in this study and 1037 (79%) of them presented with SIMI. The multivariate Cox regression analysis in all septic patients revealed that SIMI was independently associated with 28-day mortality of septic patients. The risk factors of diabetes, Apache II score, mechanical ventilation, vasoactive support, Troponin T and creatinine were included in the model and a nomogram was constructed based on the model. The C-index, AUC, NRI, IDI, calibration plotting and DCA showed that the performance of the nomogram was better than the single SOFA score and Troponin T.

**Conclusion:**

SIMI is related to the 28-day mortality of septic patients. The nomogram is a well-performed tool to predict accurately the 28-day mortality in patients with SIMI.

**Supplementary Information:**

The online version contains supplementary material available at 10.1186/s12871-023-02189-8.

## Introduction

Sepsis is the most common disease and remains the leading cause of death in intensive care unit (ICU) patients, resulting in a huge health burden in worldwide [1, 2]. Patients present with multiple life-threatening organ dysfunction in dysregulated response to infection [3]. 70% of septic patients had cardiovascular dysfunction characterized by cardiac biomarker elevation [4]. A post-mortem necropsy study for patients with sepsis or septic shock demonstrated more than half of the patients had myocardial injury [5]. Moreover, sepsis-induced myocardial injury (SIMI) increases the mortality of patients [6]. The clinical outcomes for patients with SIMI could be improved if they are assessed early and preventive measures are taken in time.

The assessment of SIMI is mainly based on echocardiography and biomarkers. Echocardiography requires high image quality and operation skills, and errors in operators are difficult to avoid [7]. While the results of studies regarding the differences in biomarkers were inconsistent [8]. Acute Physiology and Chronic Health Evaluation II (APACHE II) score and Sequential Organ Failure Assessment (SOFA) score have good efficacy in the assessment of the severity of the disease and prognosis of patients with sepsis [9], however, they have no specificity in SIMI. Therefore, our study aims to develop a nomogram for predicting the 28-day mortality of patients withSIMI based on Medical Information Mart for Intensive Care (MIMIC-IV) database.

## Methods

### Study design and study population

This retrospective observational study was conducted based on the Medical Information Mart for Intensive Care IV (MIMIC-IV, version 2.0) database from 2008 to 2019 [10], in which patients were identified. The inclusion criteria were: (1) diagnosed sepsis according to sepsis-3 definition [3]; (2) aged 18–80 years. The following exclusion criteria were applied: (1) other cardiovascular diseases (including coronary diseases, cardiomyopathy caused by non-sepsis, myocarditis, chronic obstructive pulmonary disease, chronic heart failure and valvular disease); (2) without an examination of Troponin T. For the eligible patients, the following data were retrospectively collected: (1) demographic characteristics including age, gender, and weight; (2) comorbidity; (3) the severity of illness including Acute Physiology Age and Chronic Health Evaluation (APACHE II) score and sequential organ failure assessment (SOFA) score; (4) organ failure was recorded by mechanical ventilation, continuous renal replacement therapy (CRRT) and vasoactive support within 24 h since ICU admission; (5) the first laboratory data since ICU admission; (6) 28-day mortality, length of ICU stay and length of hospital stay. The primary outcome was 28-day mortality.

The Sepsis-3 was defined as patients with life-threatening organ dysfunction caused by infection and SOFA score ≥ 2 from baseline [3]. There have been no formalized or consensus definition for SIMI. Troponin T is used to evaluatemyocardial injury in septic patients for clinical practice and studies have proven that Troponin T was associated with mortality in sepsis patients [8]. The 99th percentile of upper reference limit value for Troponin T is 0.01 ng/mL in this center, and SIMI was defined as the Troponin T within 24 h ≥ 0.01 ng/ml in this study [6].

### Source of data and Ethics approval

MIMIC-IV is an open-source clinical database that contains comprehensive and reliable clinical information of patients who visited the intensive care unit in the Beth Israel Deaconess Medical Center between 2008 and 2019, including disease diagnoses, demographic data, laboratory results, detailed treatment and outcomes information [10]. After obtaining access to the MIMIC-IV database, the data was extracted freely by researchers and Structured Query Language (SQL) with Navicat Premium was used to extract the data. In this study, Dr.KZ, X was approved to have access to the data from MIMIC-IV. Since MIMIC-IV was approved by the Massachusetts Institute of Technology (Cambridge, MA, USA) and Beth Israel Deaconess Medical Center (Boston, MA, USA), and informed consent was obtained. Moreover, all the patient information was covered up, ethical review was not required.

### Data management and statistical analysis

Statistical analyses were performed using R version 4.2.0 software. Firstly, the multiple imputation method was used to fill in missing data, and variables with more than 20% missing data were deleted [11]. Shapiro-Wilk tests were performed to assess the distribution of variables. For continuous data, variables with normal distribution were reported as mean ± standard deviation, and variables with skewed distribution were reported as median and interquartile range. Count data were expressed as numbers and percentages. Student′s t tests and Mann-Whitney *U* test tests were used to compare the continuous data. The chi-squared tests were applied to compare the categorical variables between the two groups. Prognostic factors were assessed by univariate analyses. To adjust for potential confounders, variables related to 28-day death in univariate analysis (*p* < 0.1) were entered into analysis by backward stepwise Cox proportional hazards regression modeling. And Akaike′s information criterion was used to select the final model. Interaction tests were further performed between the prognostic variables. Variance inflation factor (VIF) and Pearson’ s correlation coefficients to assess collinearity for continuous data. VIF > 5 was considered to indicate collinearity [12], and we also included variables with a correlation coefficient < 0.5 [13]. The 28-day survival was estimated using the Kaplan-Meier and compared by log-rank test.

Patients enrolled with SIMI were randomly distributed into the training cohort and validation cohort at the ratio of 7:3. And a nomogram for predicting 28-day mortality of SIMI was constructed in training cohort according to Occam′s Law, in which fewer variables should be included to achieve the aim [14] Concordance index (C-index) and the area under the receiver operating characteristic curve (AUC) were performed to assess the effectiveness of the nomogram [15], the integrated discrimination improvement (IDI) and the net reclassification improvement (NRI) were carried out to evaluate the accuracy of nomogram [16, 17]. Calibration plots by bootstrap method were used to evaluate consistency between the predicting probability and the actual probability, and decision curve analysis (DCA) was used to assess the net benefit of nomogram at different threshold probabilities [18].

## Results

### Characteristics of included participants

**Fig. 1:**
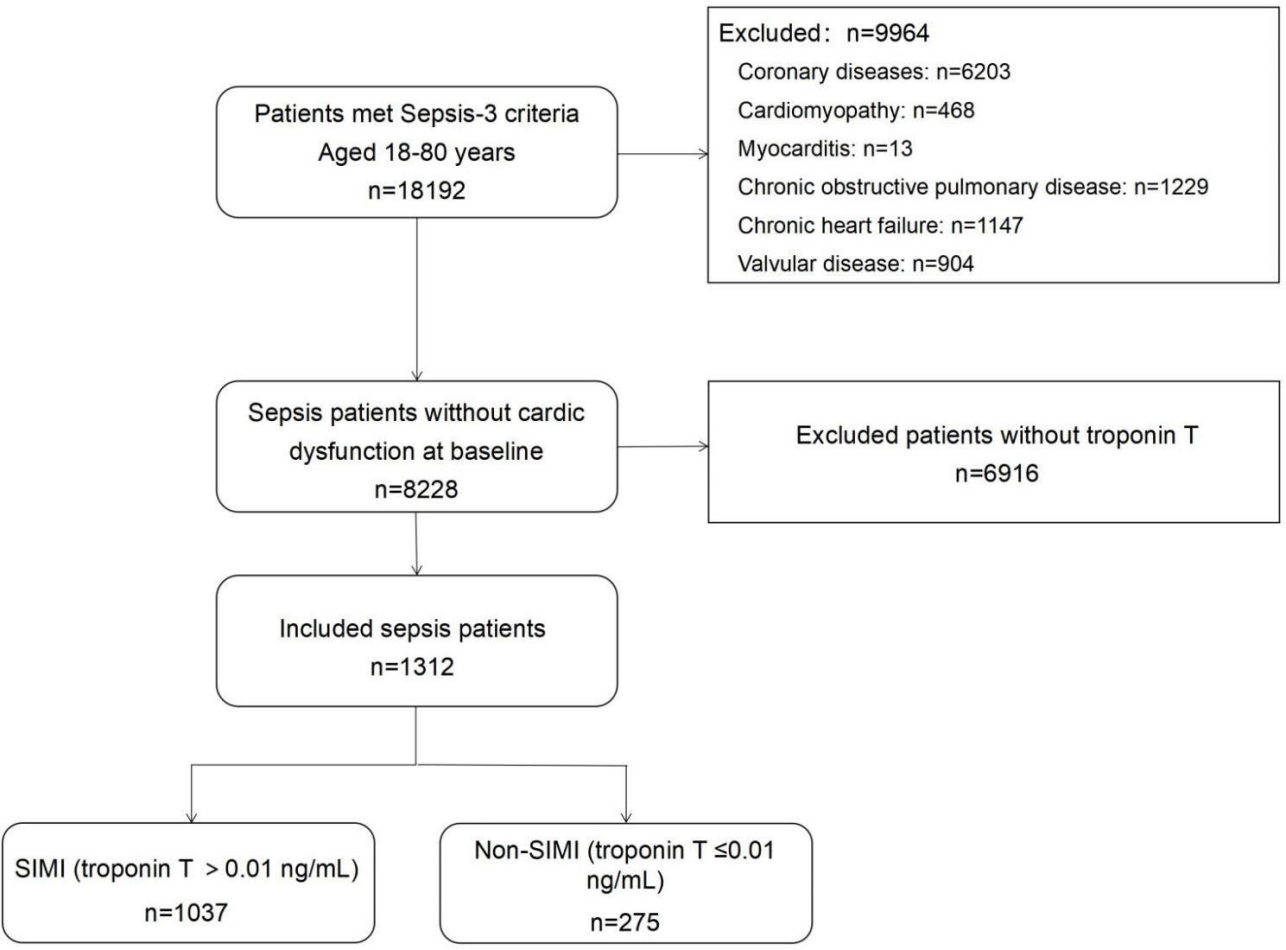
Flow chart

The flowchart of the study population with inclusions and exclusions was given in Fig. 1. 1312 patients were included in the study and 1037 (79.0%) patients presented with SIMI. The demographic and clinical characteristics of SIMI and Non-SIMI were provided in Additional file 1: Table S1. The SICM had higher 28-day mortality compared to Non-SICM patients (32.6% vs. 17.1%, *p* < 0.001). A Cox regression multivariable analysis was performed in all septic patients, and SIMI was independently associated with the 28-day mortality of septic patients (Additional file 2: Table S2). The Kaplan-Meier curve showed that the 28-day mortality was significantly higher in patients with SIMI (Additional file 3: Figure S1).

Patients included were randomly divided into training cohort (n = 727) and validating cohort (n = 310) in the ratio of 7:3, and demographic data and clinical information were comparable between the two cohorts (Additional file 4: Table S3). In the training cohort, 237 (32.6%) patients died within 28 days, and demographic and clinical characteristics were given in Table 1. Death group had a higher proportion of diabetes than survival group (29.5% vs. 21.8%, p = 0.033). Death group had higher APACHE II score (28.0 vs. 23.0, *p* = 0.000), SOFA score (11.0 vs. 7.0, *p* = 0.000). the maximal dose of norepinephrine (3.6 µg/kg/min vs. 0.0 µg/kg/min, *p* = 0.000) and Troponin T (0.08ng/ml vs. 0.06ng/ml, *p* = 0.000) than that of survivor group. And there were more patients received mechanical ventilation (77.2% vs. 56.9%, p = 0.000), CRRT (7.2% vs. 2.2%, *p* = 0.003) and vasoactive support (81.4% vs. 49.0%, p = 0.000) in death group compared with survivors.

**Table 1:** Demographic and clinical characteristics of the training cohort

Variables	Death (n = 237)	Survival (n = 490)	ASMD	*p*
Demographics and comorbidities				
Age, years	60.3 (47.3–68.8)	59.9 (47.7–69.5)	0.001	0.843
Male gender, n (%)	145 (61.2%)	284 (58%)	0.046	0.422
Weight, kg	83.5 (70.0-96.8)	81.5 (70.0-100.0)	0.002	0.862
Hypertension, n (%)	124 (52.3%)	276 (56.3%)	0.057	0.340
Diabetes, n (%)	70 (29.5%)	107 (21.8%)	0.124	0.033
Chronic kidney disease, n (%)	20 (8.4%)	56 (11.4%)	0.071	0.246
Infection site, n (%)				0.136
Lung	74 (31.2%)	181 (36.9%)		
Gastrointestinal tract	12 (5.1%)	26 (5.3%)		
Urinary	33 (13.9%)	122 (24.9%)		
Skin and soft tissue	7 (3%)	29 (5.9%)		
APACHE II score ^a^	28.0 (21.0–33.0)	23.0 (17.0–28.0)	0.068	< 0.001
SOFA score ^a^	11.0 (7–14.0)	7.0 (5.0-10.3)	0.160	< 0.001
Organ failures ^b^				
Mechanical ventilation, n (%)	183 (77.2%)	279 (56.9%)	0.312	< 0.001
CRRT, n (%)	17 (7.2%)	11 (2.2%)	0.166	0.003
Vasoactive support, n (%)	193 (81.4%)	240 (49.0%)	0.512	< 0.001
Maximal dose of norepinephrine (µg/kg/min)	3.6 (0.0–8.0)	0.0 (0.0-2.3)	0.190	< 0.001
Maximal dose of epinephrine (µg/kg/min)	0.0 (0.0–0.0)	0.0 (0.0–0.0)	0.162	< 0.001
Laboratory tests ^b^				
Troponin T (ng/ml)	0.08 (0.03–0.28)	0.06 (0.03–0.14)	0.142	0.010
WBC (k/ul)	12.6 (8.5–17.4)	13.3 (8.6–18.4)	0.008	0.389
Hemoglobin (g/dl)	11.6 (9.7–13.9)	11.2 (9.5–13.3)	0.023	0.383
Platelet (k/uL)	147.0 (92.0-218.0)	164.0 (101.0-226.3)	0.001	0.189
Creatinine (mg/dl)	1.5 (1.0-3.3)	1.3 (0.9–2.4)	0.046	0.092
Clinical outcomes				
Length of ICU stay (days)	3.8 (2.2–7.9)	4.1 (2.4–9.7)	0.035	0.085
Length of hospital stay (days)	4.9 (1.9–11.6)	11.9 (6.8–21.2)	0.047	< 0.001

Data are expressed as mean ± SD, Median (interquartile range) or number (%). *APACHE* Acute Physiology Age and Chronic Health Evaluation, *SOFA* sequential organ failure assessment, *CRRT* continuous renal replacement therapy, *ICU* intensive care unit, *ASMD* absolute standardized mean difference

^a^ Apache II score and SOFA score were calculated on the first 24 h since ICU admission.

^b^ Organ failures information and laboratory tests were recorded the first result of patients′ ICU stay

### Screening for predictive factors and Nomogram development in the training set

**Fig. 2:**
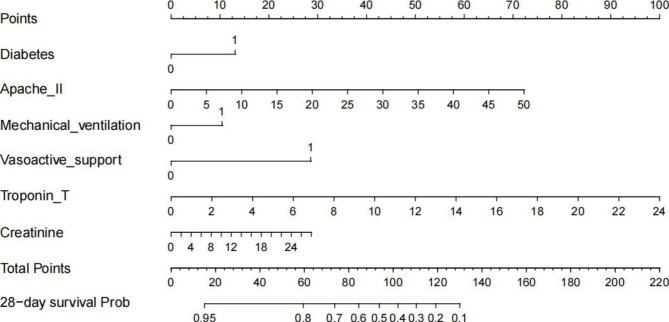
Nomogram for predicting the 28-day survival probability of patients with SIMI. When using it, drawing a vertical line from each variable upward to the points and then recording the corresponding points (i.e., “Vasoactive support = 1” =20 points). The point of each variable was then summed up to obtain a total score that corresponds to a predicted probability of 28-day survaval at the bottom of the nomogram

Prognostic factors were assessed firstly by univariate Cox analyses (Additional file 5: Table S4). The prognostic factors of SIMI identified by multivariate Cox regression were shown in Table 2. And there was no significant interactions between variables was found (*p* > 0.05). In our analyses, all VIF values were less than 5, and correlation between continuous variables was provided in Additional file 6: Figure S2, indicating that collinearity was not observed in the Cox regression model. A comprehensive evaluation of the variables was performed and we established a prognostic model according to Occam′s razor, which including diabetes (hazard ratio [HR] 1.54, 95% confidence interval [CI] 1.12–2.12), APACHE II score (HR 1.05, 95%CI 1.03–1.07), Mechanical ventilation (HR 1.41, 95%CI 1.02–1.95), Vasoactive support (HR 2.57, 95%CI 1.81–3.67), Troponin T (HR 1.15, 95%CI 1.08–1.22) and Creatinine (HR 1.03, 95%CI 0.99–1.08). Based on the model, a nomogram was constructed to predict the 28-day mortality of SIMI (Fig. 2).

**Table 2:** Multivariate Cox regression analysis for 28-day mortality in patients with SIMI

Variables	HR	95%CI	*p*
Diabetes	1.54	1.12–2.12	0.008
APACHE II score	1.05	1.03–1.07	< 0.001
Mechanical ventilation	1.41	1.02–1.95	0.037
Vasoactive support	2.57	1.81–3.67	< 0.001
Troponin T (ng/ml)	1.15	1.08–1.22	< 0.001
Creatinine (mg/dl)	1.03	0.99–1.08	0.121

*APACHE* Acute Physiology Age and Chronic Health Evaluation

### Validation of the prediction nomogram

**Fig. 3:**
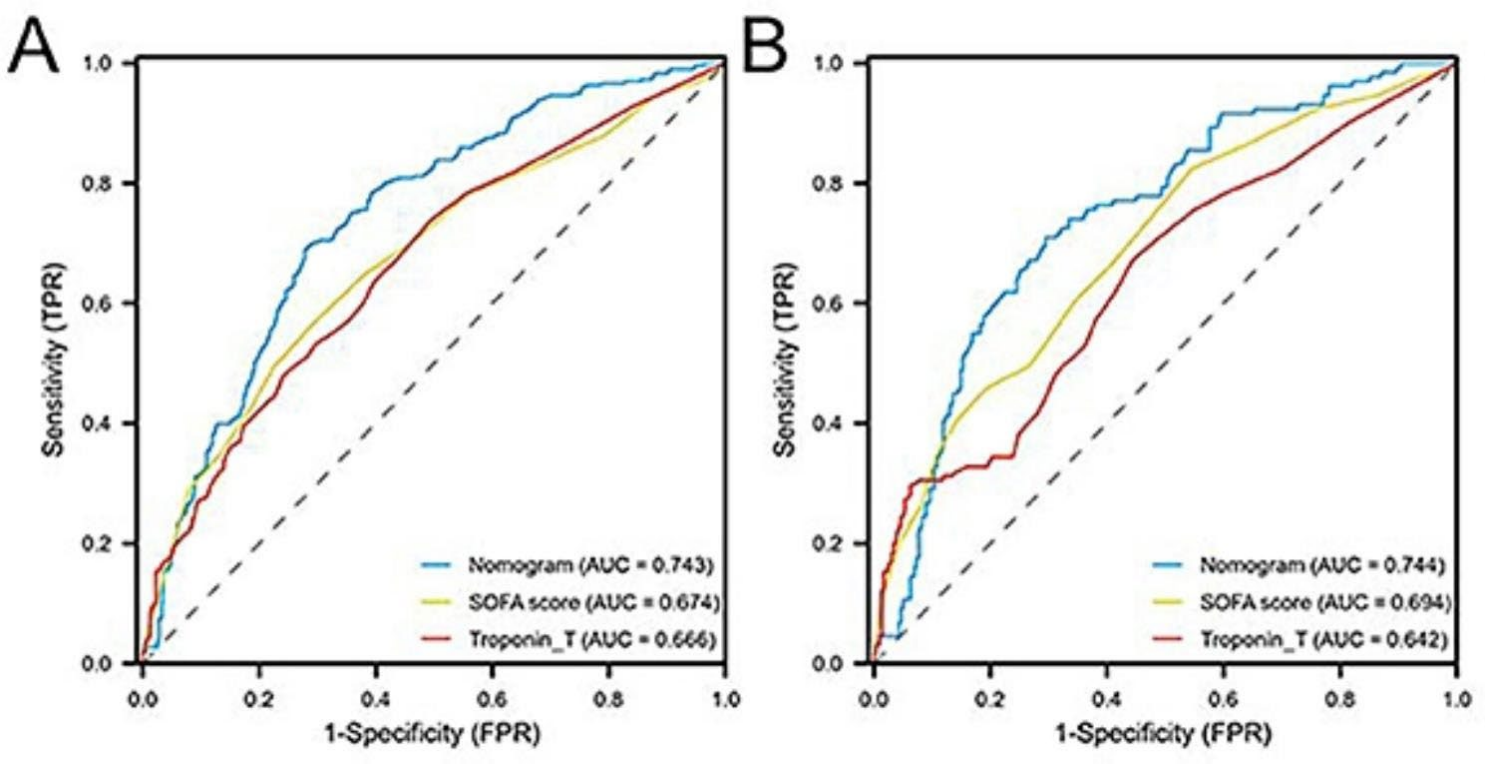
ROC curve and AUROC of Nomogram, SOFA score and Troponin T in training cohort (**A**) and validation cohort (**B**). The AUROC of nomogram is higher than that of SOFA and Troponin T in both cohorts

AUROC was used to evaluate the performance of the nomogram. As given in Fig. 3; Table 3, the AUROC of the nomogram for 28-day survival probabilities were higher than the single SOFA score and Troponin T in the training cohort (0.743 vs0.674, 0.743 vs. 0.666, respectively), as well as in the validation cohort (0.744 vs. 0.694, 0.744 vs. 0.642, respectively), indicating that the nomogram had better discrimination than SOFA score and Troponin T in predicting 28-day mortality of SIMI patients. Moreover, the NRI of nomogram was 30.67% (95% CI 23.90%-54.57%) and 41.20% (95% CI 36.05–46.35%) in the training and validation cohorts, respectively. And IDI of nomogram was significantly higher than that of the SOFA score and Troponin T in the training cohort, as well as in the validation cohort (Table 3).

**Table 3:** The AUROC and IDI of Nomogram, SOFA score and Troponin T in training cohort and validation cohort

Predictive Model		AUROC	*p* ^a^	IDI	*P*
Training cohort	Nomogram	0.743 (0.716–0.770)			
	SOFA score	0.674 (0.641–0.707)	< 0.001	10.31% (8.31%-12.31)	< 0.001
	Troponin T	0.666 (0.629–0.703)	< 0.001	9.93% (7.83%-12.03)	< 0.001
Validation cohort	Nomogram	0.744 (0.691–0.797)			
	SOFA score	0.694 (0.663 − 0.625)	< 0.001	7.05% (4.25-9.85%)	< 0.001
	Troponin T	0.642 (0.589–0.695)	< 0.001	8.23% (5.23-11.23%)	< 0.001

*AUROC* area under the receiver operating characteristic curve, *IDI* integrated discrimination improvement, *SOFA* sequential organ failure assessment, *IDI* integrated discrimination improvement

The *P* value was drew by comparing the results of Nomogram with SOFA score and Troponin T, respectively

^a^ The comparisons of AUROC between models were performed by Delong’s test

**Fig. 4:**
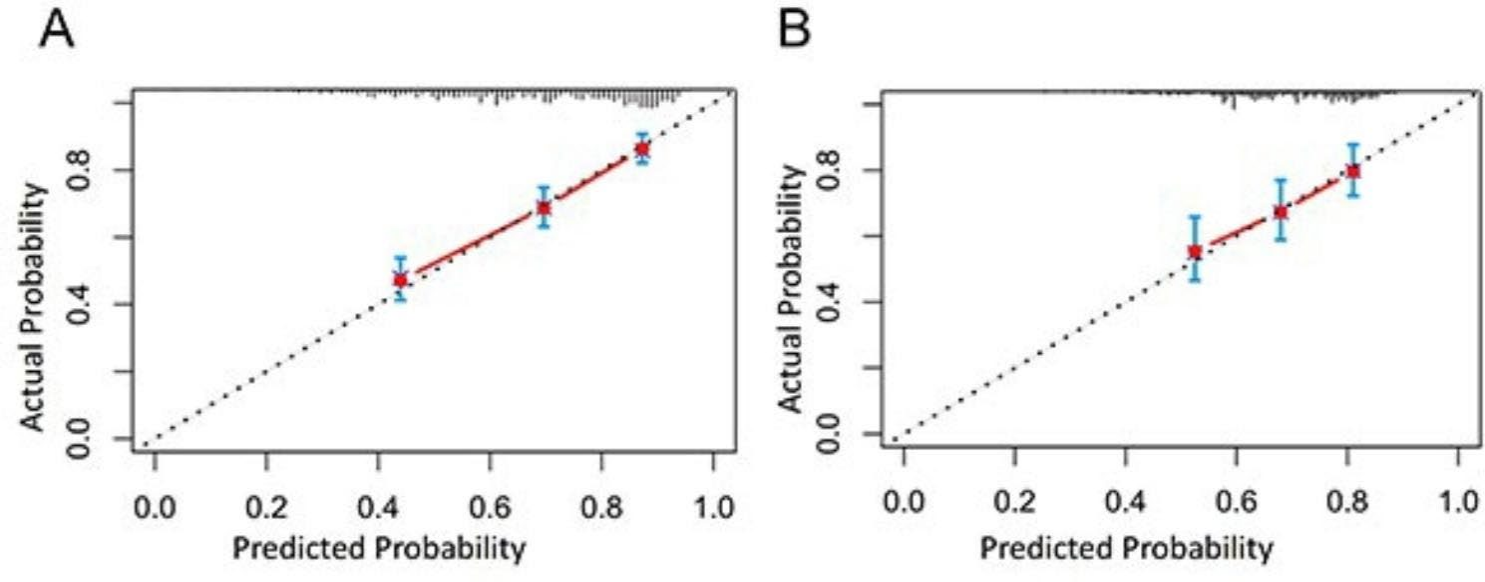
Calibration curves in training cohort (**A**) and validation cohort (**B**). In both cohorts, a good conformity between observation and prediction was observed

Model calibration, the agreement between observed outcomes and predictions, was assessed by Hosmer-Lemeshow test and calibration plots in our study. The calibration curve was conducted using the bootstrap method in both the training and validation sets. And a good conformity between predictions and observations in the calibration plot was observed in both sets (Fig. 4).

**Fig. 5:**
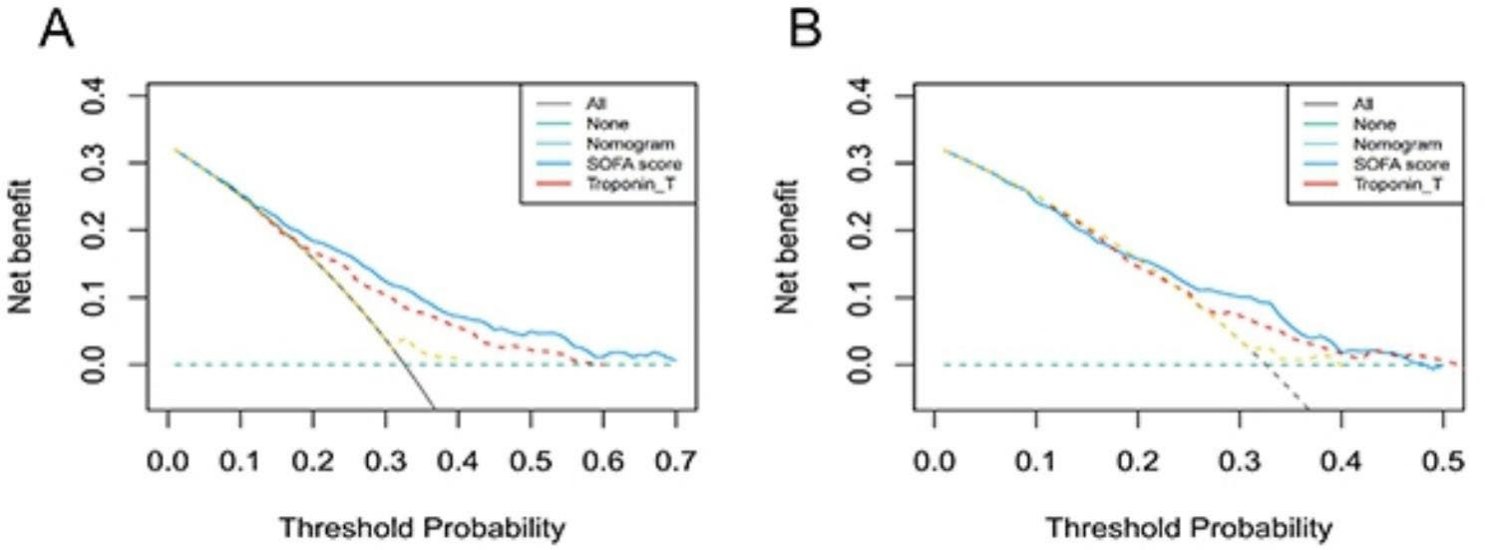
The DCA curve of medical intervention in patients with the Nomogram, SOFA score, and Troponin T in training cohort (**A**) and validation cohort (**B**)

DCA was to evaluate the clinical benefits of the nomogram model in the study. The results of DCA showed that significant benefit of the nomogram model than SOFA score and Troponin T when the probability threshold was between 0.06 and 0.75 in the training cohort (Fig. 5A). And when the threshold probability was > 0.07, the model to predict the 28-day mortality of study patients could provide a greater net benefit than SOFA score and Troponin T in the validation set (Fig. 5B).

## Discussion

In this retrospective analysis, Cox regression was performed to identify risk factors using the MIMIC IV database and predictors associated with the 28-day mortality of patients with SIMI in ICU. The identified risk factors, including diabetes, Apache II score, mechanical ventilation, vasoactive support, Troponin T and creatinine, were integrated into the most suitable prediction model, which was then presented in the form of a prediction nomogram. And this is the first to develop a visualized model for predicting the 28-day mortality of patients with SIMI.

Myocardial injury is the most common complication of organ dysfunction in patients with sepsis. Research indicates that more than 50% of patients with sepsis experience varying degrees of myocardial injury [19]. It was reported that SIMI increased the mortality of septic patients [20]. And an increasing number of studies have demonstrated the high prevalence and high fatality rate of SIMI [21–23]. However, the awareness and treatment of SIMI remain challenging because the recommendations of guidelines all depend on sepsis, not SIMI. Therefore, comprehensive assessments of the true risk of death in patients with SIMI and objective evaluation of the risk-benefit ratio of medical interventions are of major importance for clinicians to facilitate optimal medical decisions for patients with SIMI. And predictive nomograms play a crucial role in improving risk stratification for SIMI. A nomogram can be obtained by calculating the scores corresponding to each indicator based on the first row, and adding up each point to obtain a final total points. Finally, the 28-day probability can be determined based on the final row. And only 5 indicators (including diabetes, Apache II score, mechanical ventilation, vasoactive support, and Troponin T on the first 24 h since ICU admission) of SICM patients are necessary.

In our study, 79.0% of all septic patients presented SIMI. The incidence of SICM was higher than in previous reports [24, 25]. One important reason is that different diagnostic approaches to identify SIMI patients have been used. There are no diagnostic criteria for SIMI. Transthoracic echocardiography is the main method to evaluate the SIMI in clinical practice [7], even speckle-tracking echocardiographic strain analysis [26]. However, echocardiography has certain limitations as an imaging diagnostic method for SIMI due to variations in measurement results resulting from differences in sonographers’ technical expertise. On the other hand, biomarkers have predictive value for SIMI patients, including routine Troponin and novel markers, however, the correlation between many novel biomarkers and sepsis-induced myocardial injury is controversial [27, 28]. Troponin T is a sensitive marker of myocardial damage for septic patients, which is simple and easy to acquire in retrospective research. The results of our study were similar to that of Vallabhajosyula et al., who reported that 78% of septic patients had higher Troponin T, and Troponin T is related to the mortality of these patients [6]. The results in our study showed that the mortality of SIMI identified by Troponin T was significantly higher than that of non-SIMI. It proved that Troponin T has good power to distinguish SIMI and Non-SIMI.

The elevation of Troponin T in the plasma of patients with sepsis is associated with myocardial injury [22]. Studies suggest that the increase in Troponin levels in sepsis-induced myocardial injury is not caused by myocardial cell death due to ischemia but rather by myocardial cell membrane permeability changes, Troponin leakage resulting from myocardial inhibitory factors, oxidative stress, and inflammatory response [29]. A retrospective study found a close relationship between elevated cTnT and the severity and mortality of sepsis, increasing the risk of death in sepsis patients [30]. However, another study suggested that there was no significant difference between cTnT levels assessed upon admission and 28-day mortality [31]. The varying results may be attributed to the critical threshold for cTnT elevation or differences in the timing of Troponin measurement. Masson et al. proposed that early changes in Troponin levels may serve as better predictors of poor prognosis than static Troponin levels [32]. The predictive nomogram developed in this study exhibited superior predictive performance for both 28-day and compared to Troponin T. Furthermore, to verify its clinical effectiveness, the same results were repliated in the validation cohort, demonstrating that the current nomogram-guided intervention provided greater net benefits than Troponin T.

SOFA score has proven to be a useful tool for predicting short-term mortality in septic patients [3, 33], but its appropriateness for SIMI remains unclear. Previous studies have found that the SOFA scores of severely ill patients with different sources of infection vary [34], and the SOFA score has been used to assess subsequent infection risk in severe trauma patients [35]. Additionally, the SOFA score has demonstrated better specificity in predicting the ICU hospitalization rate and mortality of patients with acute pancreatitis compared to other prognostic scores such as the bedside acute pancreatitis severity index and APACHE II score [36]. Moreover, the application of the SOFA score has shown better predictive value for the 28-day mortality risk of patients with acute infection compared to the rapid sequential organ failure score and systemic inflammatory response syndrome score [37]. In our study, multivariate Cox regression model analysis by stepwise backward was performed using the minimum AIC as the criterion. And SOFS score was eliminated in the final model The nomogram developed in this study exhibited superior predictive performance for both 28-day and 90-day mortality compared to the SOFA score and demonstrated acceptable differentiation and calibration. Furthermore, to verify its clinical effectiveness, decision curve analysis was utilized to evaluate the benefits and costs of medical interventions for patients with sepsis-induced myocardial injury under nomogram guidance. The results indicated that the nomogram-guided intervention provided greater net benefits than the SOFA score.

Recent studies have focused on patients with septic right ventricular dysfunction [38]. Invasive mechanical ventilation is a significant cause of right ventricular dysfunction [39]. Positive pressure ventilation leads to increased pulmonary vascular resistance, resulting in right ventricular dysfunction due to excessive alveolar expansion. In this study, the use of ventilation was identified as an independent risk factor for in-hospital mortality in sepsis patients.

This study has several limitations. Firstly, it is a single-center retrospective study, and the established model lacks evidence for generalizability. Secondly, while patients with preexisting cardiac disease were excluded, it was difficult to exclude patients who developed new or concealed cardiovascular events during hospitalization. Finally, the absence of formal diagnostic criteria for sepsis-induced myocardial injury remains a significant challenge. Troponin T is highly sensitive for diagnosing sepsis-induced myocardial injury but lacks specificity. Once formal diagnostic criteria are established, the nomogram model needs to be validated externally in the future.

## Conclusion

A prediction nomogram model included 5 conventional clinical parameters: Apache II score, mechanical ventilation, vasoactive support, Troponin T and creatinine. This model can predict accurately the 28-day mortality in patients with SIMI, and internal validation proved the robustness and performance of that. The application of this model can improve the prognostic evaluation and treatment strategies to decrease the 28-day mortality for patients with SIMI.

## Electronic supplementary material

Below is the link to the electronic supplementary material.

**Table S1** Demographic and clinical characteristics of the SIMI and Non-SIMI

**Table S2** Cox regression analysis for 28-day mortality in all septic patients (n=1312)

**Additional file 3: Figure S1** The Kaplan-Meier?s survival estimated of the 28-day survival probability of SIMI and Non-SIMI patients.

**Table S3** Demographic and clinical characteristics of the training cohort and validation cohort

**Table S4** Univariate Cox analyses for28-day mortality of patients with SIMI in training cohort

**Additional file 6: Figure S2** The correlation between continuous variables which were associated with the 28-day mortality of SIMI patients in the multivariable Cox regression.

## Data Availability

Data presented in this study were extracted by Xu, the raw datasets of this study may be available from the corresponding author upon reasonable request.

## List of abbreviations

SIMI: Sepsis-induced myocardial injury
APACHEII: Acute Physiology and Chronic Health Evaluation II
SOFA: Sequential Organ Failure Assessment
CRRT: Continuous renal replacement therapy
AUROC: Area Under the Receiver Operating Characteristic
NRI: Net reclassification improvement
IDI: Integrated discrimination improvement
DCA: Decision Curve Analysis
ICU: Intensive Care Unit
MIMIC-IV: Medical Information Mart for Intensive Care IV
HR: Hazard ratio
CI: Confidence Interval
