# Supplementary material for: Predictors and nomogram of in-hospital mortality in sepsis-induced myocardial injury: a retrospective cohort study
Source: BMC Anesthesiol. 2023 Jul 7;23:230. doi: 10.1186/s12871-023-02189-8 (PMC10327384; doi:10.1186/s12871-023-02189-8)
Supplement: Supplementary file 1 — Table S1 Demographic and clinical characteristics of the SIMI and Non-SIMI [file 12871_2023_2189_MOESM1_ESM.docx]

| **Table S1 Demographic and clinical characteristics of the SIMI and Non-SIMI** | | | | | |
| --- | --- | --- | --- | --- | --- |
| Variables | All  (n=1312) | SIMI  (n=1037) | Non-SIMI  (n=275) | ASMD | *p* |
| Variables |  |  |  |  |  |
| Demographics and comorbidities |  |  |  |  |  |
| Age, years | 60.4 (48.9-69.) | 60.3 (48.4-69.0) | 62.1 (53.0-71.9) | 0.017 | 0.302 |
| Male gender, n (%) | 979 (84.4%) | 602 (58.1%) | 172 (62.6%) | 0.066 | 0.384 |
| Weight, kg | 81.4 (69-97.5) | 81.5 (69.0-97.7) | 81.3 (70.0-96.6) | 0.002 | 0.962 |
| Hypertension, n (%) | 651 (56.1%) | 575 (55.4%) | 170 (61.8%) | 0.091 | 0.211 |
| Diabetes, n (%) | 319 (27.5%) | 286 (27.6%) | 74 (26.8%) | 0.012 | 0.915 |
| Chronic kidney disease, n (%) | 129 (11.1%) | 111 (10.7%) | 40 (14.6%) | 0.084 | 0.223 |
| Infection site, n (%) |  |  |  |  | 0.485 |
| Lung | 402 (34.7%) | 363 (35.0%) | 85 (30.9%) |  |  |
| Gastrointestinal tract | 65 (5.6%) | 58 (.5.6%) | 16 (5.7%) |  |  |
| Urinary | 251 (21.6%) | 222 (21.4%) | 65 (23.6%) |  |  |
| Skin and soft tissue | 71 (6.1%) | 61 (5.9%) | 22 (8.1%) |  |  |
| APACHE II score ^a^ | 24.0 (18.0-30.0) | 25.0 (19.0-30.5) | 21.0 (16.0-27.0) | 0.034 | 0.001 |
| SOFA score ^a^ | 8.0 (5.0-12.0) | 8.0 (5.0-12.0) | 7.0 (5.0-10.0) | 0.058 | 0.003 |
| Organ failures ^b^ |  |  |  |  |  |
| Mechanical ventilation, n (%) | 719 (62.0%) | 661 (59.4%) | 161 (58.5%) | 0.175 | 0.001 |
| CRRT, n (%) | 46 (4.0%) | 43 (4.1%) | 7 (2.4%) | 0.068 | 0.469 |
| Vasoactive support, n (%) | 688 (59.3%) | 616 (59.4%) | 161 (58.5%) | 0.012 | 0.847 |
| Maximal dose of norepinephrine (µg/kg/min) | 0.0 (0-4.4) | 0.0 (0-4.7) | 0.0 (0-2.3) | 0.081 | 0.482 |
| Maximal dose of epinephrine (µg/kg/min) | 0.0 (0.0-0.0) | 0.0 (0.0-0.0) | 0.0 (0.0-0.0) | 0.059 | 0.075 |
| Laboratory tests ^b^ |  |  |  |  |  |
| Troponin T (ng/ml) | 0.05 (0.02-0.14) | 0.06 (0.03-0.16) | 0.01 (0.01-0.01) | 0.379 | <0.001 |
| WBC (k/ul) | 11.3 (9.5-13.4) | 11.4 (9.5-13.5) | 10.8 (9.1-12.6) | 0.002 | 0.011 |
| Hemoglobin (g/dl) | 12.5 (8.5-18.2) | 12.5 (8.6-18.1) | 12.7 (8.4-20.0) | 0.099 | 0.781 |
| Platelet (k/uL) | 156.0 (98.0-223.0) | 155.0 (97.0-221.5) | 158.0 (105.0-232.0) | <0.001 | 0.380 |
| Creatinine (mg/dl) | 1.3 (0.9-2.5) | 1.3 (0.9-2.6) | 1.2 (0.8-2) | 0.112 | 0.033 |
| Clinical outcomes |  |  |  |  |  |
| Died within 28 days, n (%) | 359 (30.9%) | 338 (32.6%) | 47 (17.1%) | 0.258 | <0.001 |
| Length of ICU stay (days) | 4.0 (2.2-8.9) | 4.0 (2.2-9.0) | 4.0 (2.2-7.9) | 0.001 | 0.463 |
| Length of hospital stay (days) | 9.5 (4.8-18.3) | 9.6 (4.7-18.2) | 8.8 (4.8-19.3) | 0.001 | 0.853 |
| Data are expressed as mean±SD, Median (interquartile range) or number (%). *APACHE* Acute Physiology Age and Chronic Health Evaluation, *SOFA* sequential organ failure assessment, *CRRT* continuous renal replacement therapy, *ICU* intensive care unit, *ASMD* absolute standardized mean difference  ^a^ Apache II score and SOFA score were calculated on the first 24h since ICU admission.  ^b^ Organ failures information and laboratory tests were recorded the first result of patients′ ICU stay | | | | | |
