# Supplementary material for: Predictors and nomogram of in-hospital mortality in sepsis-induced myocardial injury: a retrospective cohort study
Source: BMC Anesthesiol. 2023 Jul 7;23:230. doi: 10.1186/s12871-023-02189-8 (PMC10327384; doi:10.1186/s12871-023-02189-8)
Supplement: Supplementary file 2 — Table S2 Cox regression analysis for 28-day mortality in all septic patients (n=1312) [file 12871_2023_2189_MOESM2_ESM.docx]

| **Table S2 Cox regression analysis for 28-day mortality in all septic patients (n=1312)** | | | | | | |
| --- | --- | --- | --- | --- | --- | --- |
| Univariate analysis | | | Multivariate analysis^a^ | | | |
|  |  |  | Model 1 | | Model 2 | |
| Variables | HR (95%CI) | *p* | HR (95%CI) | *p* | HR (95%CI) | *p* |
| Age, years | 1.00 (0.99-1.01) | 0.511 | - | - | - | - |
| Male gender | 1.06 (0.86-1.31) | 0.591 | - | - | - | - |
| Weight, kg | 1.00 (0.99-1.00) | 0.448 | - | - | - | - |
| Hypertension | 1.30 (1.06-1.60) | 0.014 | 1.38 (0.90-1.58) | 0.260 | 0.92 (0.74-1.14) | 0.453 |
| Diabetes | 1.30 (1.02-1.66) | 0.034 | 1.16 (0.98-1.34) | 0.340 | 0.89 (0.69-1.15) | 0.379 |
| Chronic kidney disease | 1.62 (1.10-2.38) | 0.015 | 1.22 (1.07-1.37) | 0.010 | 1.26 (0.83-1.69) | 0.404 |
| APACHE II score | 1.06 (1.04-1.07) | <0.001 | 1.07 (1.00-1.14) | <0.001 | - | - |
| SOFA score | 1.13 (1.10-1.15) | <0.001 | - | - | 1.34 (1.02-1.76) | 0.035 |
| Infection site |  |  |  |  |  |  |
| Lung | 1.00 (0.95-1.06) | 0.205 | - | - | - | - |
| Gastrointestinal tract | 0.91 (0.88-1.03) | 0.162 | - | - | - | - |
| Urinary | 1.23 (0.93-1.50) | 0.536 | - | - | - | - |
| Skin and soft tissue | 1.48 (0.88-2.48) | 0.137 | - | - | - | - |
| Mechanical ventilation | 2.29 (1.80-2.92) | <0.001 | 1.46 (1.13-1.90) | <0.001 | 1.43(0.94-2.19) | 0.098 |
| CRRT | 2.64 (1.77-3.93) | <0.001 | 1.26 (0.83-1.93) | 0.280 | 1.90 (1.42-2.54) | <0.001 |
| Vasoactive support | 2.97 (2.31-3.81) | <0.001 | 2.13 (1.62-2.79) | <0.001 | 1.76 (1.13-2.74) | 0.013 |
| SIMI (Troponin T>0.01 ng/ml) | 0.48 (0.31-0.74) | 0.001 | 1.06 (1.03-1.10) | <0.001 | 1.07 (1.04-1.10) | <0.000 |
| Hemoglobin, g/dl | 1.00 (1.00-1.01) | 0.507 | - | - | - | - |
| WBC (k/ul) | 1.05 (1.01-1.09) | 0.260 | - | - | - | - |
| Platelet (k/uL) | 1.00 (1.00-1.00) | 0.287 | - | - | - | - |
| Creatinine (mg/dl) | 1.06 (1.02-1.09) | 0.001 | 1.04 (1.00-1.09) | 0.040 | 1.07 (1.03-1.11) | <0.001 |
| *APACHE* Acute Physiology Age and Chronic Health Evaluation, *SOFA* sequential organ failure assessment, *CRRT* continuous renal replacement therapy, *HR* hazard ratios, *CI* confidence interval.  ^a^ To identify independent prognostic factors, variables related to 28-day death in univariate analysis (*p*<0.1) were entered into multivariate Cox proportional hazards regression model with forced entry. Due to the colinearity between APACHE II and SOFA (correlation coefficient=0.602, and there was no collinearity in other variables.), these variables were included separately in 2 multivariable models. | | | | | | |
