# Supplementary material for: Predictors and nomogram of in-hospital mortality in sepsis-induced myocardial injury: a retrospective cohort study
Source: BMC Anesthesiol. 2023 Jul 7;23:230. doi: 10.1186/s12871-023-02189-8 (PMC10327384; doi:10.1186/s12871-023-02189-8)
Supplement: Supplementary file 3 — Additional file 3: Figure S1 The Kaplan-Meier?s survival estimated of the 28-day survival probability of SIMI and Non-SIMI patients. [file 12871_2023_2189_MOESM3_ESM.docx]

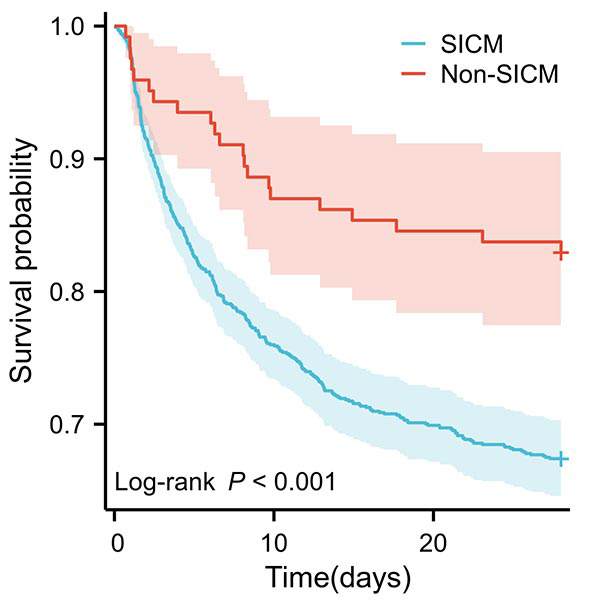


Additional file 3: Figure S1 The Kaplan-Meier′s survival estimated of the 28-day survival probability of SIMI and Non-SIMI patients.
