# Supplementary material for: Predictors and nomogram of in-hospital mortality in sepsis-induced myocardial injury: a retrospective cohort study
Source: BMC Anesthesiol. 2023 Jul 7;23:230. doi: 10.1186/s12871-023-02189-8 (PMC10327384; doi:10.1186/s12871-023-02189-8)
Supplement: Supplementary file 4 — Table S3 Demographic and clinical characteristics of the training cohort and validation cohort [file 12871_2023_2189_MOESM4_ESM.docx]

| **Table S3 Demographic and clinical characteristics of the training cohort and validation cohort** | | | | |
| --- | --- | --- | --- | --- |
| Variables | Training cohort  (n=727) | Validation cohort  (n=310) | ASMD | *p* |
| Demographics and comorbidities |  |  |  |  |
| Age, years | 60.1 (47.7-69.3) | 61.1 (49.9-68.3) | 0.006 | 0.423 |
| Male gender, n (%) | 429 (59.0%) | 173 (55.8%) | 0.046 | 0.372 |
| Weight, kg | 82.0 (70.0-99.0) | 80.0 (65.8-93.2) | 0.007 | 0.106 |
| Hypertension, n (%) | 400 (55.0%) | 175 (56.5%) | 0.020 | 0.683 |
| Diabetes, n (%) | 197 (27.1%) | 89 (28.7%) | 0.025 | 0.196 |
| Chronic kidney disease, n (%) | 76 (10.5%) | 35 (11.3%) | 0.019 | 0.742 |
| Infection site, n (%) |  |  |  | 0.461 |
| Lung | 255 (35.1%) | 108 (34.8%) |  |  |
| Gastrointestinal tract | 38 (5.2%) | 20 (6.5%) |  |  |
| Urinary | 155 (21.3%) | 67 (21.6%) |  |  |
| Skin and soft tissue | 36 (5.0%) | 25 (8.1%) |  |  |
| APACHE II score ^a^ | 24.0 (19.0-30.0) | 25.0 (19.0-31.0) | 0.011 | 0.181 |
| SOFA score ^a^ | 8.0 (5.0-12.0) | 8.0 (5.0-12.0) | 0.001 | 0.852 |
| Organ failures ^b^ |  |  |  |  |
| Mechanical ventilation, n (%) | 462 (63.5%) | 199 (64.2%) | 0.009 | 0.888 |
| CRRT, n (%) | 28 (3.9%) | 15 (4.8%) | 0.034 | 0.497 |
| Vasoactive support, n (%) | 433 (60.0%) | 183 (59.0%) | 0.008 | 0.890 |
| Maximal dose of norepinephrine (µg/kg/min) | 0.0 (0-4.7) | 0.0 (0-5.1) | 0.001 | 0.892 |
| Maximal dose of epinephrine (µg/kg/min) | 0.0 (0.0-0.0) | 0.0 (0.0-0.0) | 0.003 | 0.465 |
| Laboratory tests ^b^ |  |  |  |  |
| Troponin T (ng/ml) | 0.06 (0.03-0.15) | 0.06 (0.03-0.18) | 0.067 | 0.998 |
| WBC (k/ul) | 12.9 (8.5-18.3) | 12.2 (8.8-16.9) | 0.001 | 0.622 |
| Hemoglobin (g/dl) | 11.4 (9.6-13.5) | 11.4 (9.4-13.6) | 0.001 | 0.91 |
| Platelet (k/uL) | 158.0 (98.0-225.0) | 149.0 (90.8-213.5) | 0.001 | 0.160 |
| Creatinine (mg/dl) | 1.4 (0.9-2.6) | 1.3 (0.9-2.5) | 0.027 | 0.492 |
| Clinical outcomes |  |  |  |  |
| Died within 28 days, n (%) | 237 (32.6%) | 101 (32.6%) | 0.000 | 1.000 |
| Length of ICU stay (days) | 4 .0 (2.3-9.1) | 3.9 (2.1-9.0) | 0.004 | 0.598 |
| Length of hospital stay (days) | 9.6 (4.7-17.7) | 9.7 (5.0-20.2) | 0.007 | 0.319 |
| Data are expressed as mean±SD, Median (interquartile range) or number (%). *APACHE* Acute Physiology Age and Chronic Health Evaluation, *SOFA* sequential organ failure assessment, *CRRT* continuous renal replacement therapy, *ICU* intensive care unit, *ASMD* absolute standardized mean difference  ^a^ Apache II score and SOFA score were calculated on the first 24h since ICU admission.  ^b^ Organ failures information and laboratory tests were recorded the first result of patients′ ICU stay | | | | |
