# Supplementary material for: Predictors and nomogram of in-hospital mortality in sepsis-induced myocardial injury: a retrospective cohort study
Source: BMC Anesthesiol. 2023 Jul 7;23:230. doi: 10.1186/s12871-023-02189-8 (PMC10327384; doi:10.1186/s12871-023-02189-8)
Supplement: Supplementary file 5 — Table S4 Univariate Cox analyses for28-day mortality of patients with SIMI in training cohort [file 12871_2023_2189_MOESM5_ESM.docx]

| Table S4 Univariate Cox analyses for28-day mortality of patients with SIMI in training cohort | | | |
| --- | --- | --- | --- |
| Variables | HR | 95%CI | *P* |
| Age, years | 1.00 | 0.99-1.01 | 0.570 |
| Male gender | 0.88 | 0.88-1.48 | 0.324 |
| Weight, kg | 1.00 | 0.99-1.00 | 0.613 |
| Hypertension | 1.20 | 0.65-1.08 | 0.161 |
| Diabetes | 1.44 | 1.23-1.65 | 0.020 |
| Chronic kidney disease | 1.37 | 0.46-1.15 | 0.175 |
| APACHE II score | 1.94 | 1.75-2.13 | <0.001 |
| SOFA score | 1.88 | 1.70-2.06 | <0.001 |
| Infection site |  |  |  |
| Lung | 1.11 | 0.5-1.61 | 0.729 |
| Gastrointestinal tract | 1.89 | 0.37-0.76 | 0.175 |
| Skin and soft tissueUrinary | 1.34 | 0.57-1.28 | 0.308 |
| Urinary | 1.96 | 0.24-1.08 | 0.180 |
| Mechanical ventilation | 1.43 | 1.72-3.15 | <0.001 |
| CRRT | 1.35 | 1.76-4.74 | <0.001 |
| Vasoactive support | 1.27 | 2.68-5.16 | <0.001 |
| Hemoglobin, g/dl | 1.02 | 0.97-1.07 | 0.980 |
| WBC (k/ul) | 1.01 | 0.98-1.01 | 0.264 |
| Platelet (k/uL) | 1.00 | 1.00-1.00 | 0.145 |
| Creatinine (mg/dl) | 0.96 | 0.84-1.08 | 0.056 |
| *APACHE* Acute Physiology Age and Chronic Health Evaluation, *SOFA* sequential organ failure assessment, *CRRT* continuous renal replacement therapy, *HR* hazard ratios, *CI* confidence interval. | | | |
