# Supplementary material for: Predictors and nomogram of in-hospital mortality in sepsis-induced myocardial injury: a retrospective cohort study
Source: BMC Anesthesiol. 2023 Jul 7;23:230. doi: 10.1186/s12871-023-02189-8 (PMC10327384; doi:10.1186/s12871-023-02189-8)
Supplement: Supplementary file 6 — Additional file 6: Figure S2 The correlation between continuous variables which were associated with the 28-day mortality of SIMI patients in the multivariable Cox regression. [file 12871_2023_2189_MOESM6_ESM.docx]

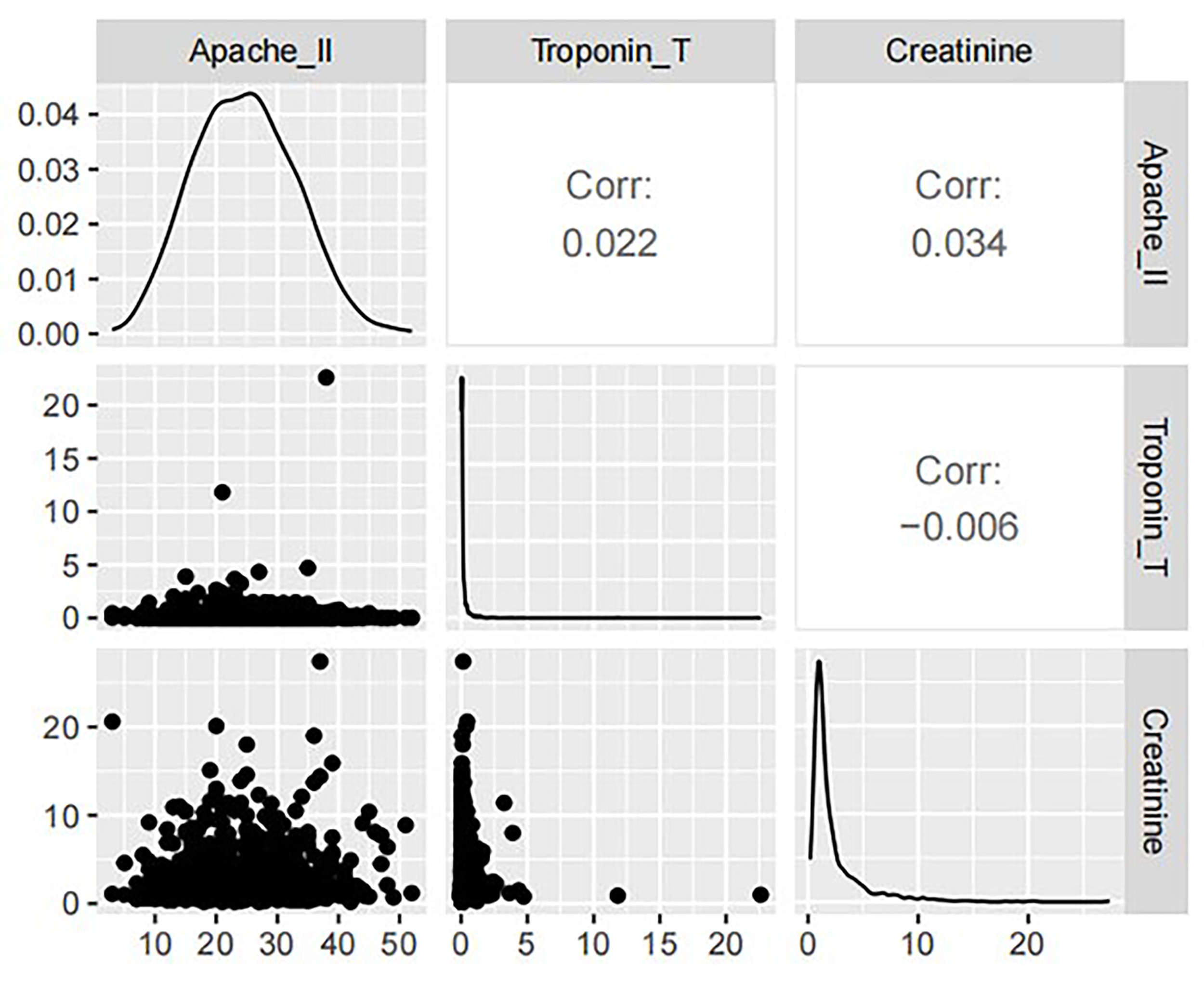


Additional file 6: Figure S2 The correlation between continuous variables which were associated with the 28-day mortality of SIMI patients in the multivariable Cox regression.
